# Re: Long term outcomes of one-stage augmentation anterior urethroplasty: a systematic review and metaanalysis

**DOI:** 10.1590/S1677-5538.IBJU.2022.0474

**Published:** 2022-11-20

**Authors:** Ke Lu, Yongchang Chen, Hengda Hu

**Affiliations:** 1 Yangzhou University Fifth Clinical Medical College Department of Urology Changshu Second People’s Hospital, Yangzhou Changshu, Suzhou China Department of Urology, Changshu Second People’s Hospital, Yangzhou University Fifth Clinical Medical College, Changshu, Suzhou, China; 2 Shanghai University of Medicine & Health Sciences Department of Urology Jinshan District Central Hospital Shanghai China Department of Urology, Jinshan District Central Hospital affiliated to Shanghai University of Medicine & Health Sciences, Jinshan District, Shanghai, China

To the editor,

We recently read an article, entitled “Long term outcomes of one-stage augmentation anterior urethroplasty: a systematic review and meta-analysis” (1). The authors summarized and concluded the long-term success of anterior augmentation urethroplasty (AU) from 10 published researchers.

Previous studies showed the success rates for augmentation urethroplasty was around 85%, yet this is an exaggerated rate as the rate declines over time according to this article. The authors claimed that the long-term success of augmentation urethroplasty seemed not as durable as reported with intermediate follow-up and showed to have continued deterioration with more than 100 months of follow-up. We are interested in the authors’ work as many doctors and patients ignore this in clinical practice. The decreasing effectiveness of AU during long-term follow-up reminds clinicians of the need to reassess this procedure and the need to inform patients about this progress.

With all due respect, there are some controversies need to be clarified. First, 10 retrospective studies were analyzed in this article. We found that patients could be recruited repeatedly in 2 researchers performed by Barbagli et al. in 2008 (2) and 2009 (3). The article published in 2009 was a brief report regarding outcomes of repair of penile urethral strictures using one-stage flap or graft urethroplasty with a maximum follow-up of 132 months. As a result, there could be duplicated data in these 2 articles.

Second, assessing the quality of included studies in meta-analyses is necessary. Generally, the Newcastle-Ottawa Scale is one of the most popular tools applied in non-randomized studies. Even if all included studies were observational studies, the authors did not give a detailed evaluation, which could undermine the rigorousness of this research.

Third, a funnel plot is not necessary to detect publication bias when there were less than 10 researchers, as symmetries are difficult to tell on this occasion. It would be perfect if some other tools, such as Egger test, had been applied in the analysis of publication bias.

Finally, the authors insightful work will inspire more similar studies and we are thankful for their contributions.

The Author
